# Safety and Survival Outcomes of ^177^Lu-Prostate-Specific Membrane Antigen Therapy in Patients with Metastatic Castration-Resistant Prostate Cancer with Prior ^223^Ra treatment: The RALU Study

**DOI:** 10.2967/jnumed.122.264456

**Published:** 2023-04

**Authors:** Kambiz Rahbar, Markus Essler, Kim M. Pabst, Matthias Eiber, Christian la Fougère, Vikas Prasad, Philipp Rassek, Ergela Hasa, Helmut Dittmann, Ralph A. Bundschuh, Wolfgang P. Fendler, Milena Kurtinecz, Anja Schmall, Frank Verholen, Oliver Sartor

**Affiliations:** 1 Department of Nuclear Medicine, University of Münster Medical Center, Münster, Germany;; 2 Department of Nuclear Medicine, University Hospital Bonn, Bonn, Germany;; 3 Department of Nuclear Medicine, German Cancer Consortium (DKTK) University Hospital Essen, Essen, Germany;; 4 Department of Nuclear Medicine, Technical University of Munich, Munich, Germany;; 5 Department of Nuclear Medicine and Clinical Molecular Imaging, University Hospital Tübingen, Tübingen, Germany;; 6 Department of Nuclear Medicine, University of Ulm, Ulm, Germany;; 7 International Centers for Precision Oncology Foundation, Ravensburg, Germany;; 8 Department of Nuclear Medicine, Medical Faculty, University of Augsburg, Augsburg, Germany;; 9 Bayer HealthCare Pharmaceuticals, Whippany, New Jersey;; 10 Bayer Consumer Care, Basel, Switzerland; and; 11 Tulane Cancer Center, Tulane Medical School, New Orleans, Louisiana

**Keywords:** targeted α-therapy, ^223^Ra, ^177^Lu-PSMA, metastatic castration-resistant prostate cancer, real-world practice

## Abstract

The radium lutetium (RALU) study evaluated the feasibility of sequential α- and β-emitter use in patients with bone-predominant metastatic castration-resistant prostate cancer. **Methods:** This preplanned interim retrospective analysis investigated safety and survival outcomes with ^177^Lu-PSMA in patients treated with prior ^223^Ra. **Results:** Forty-nine patients were evaluated. Patients received a median of 6 ^223^Ra injections; 59% of patients received at least 4 ^177^Lu-PSMA cycles. Most (69%) patients received at least 4 life-prolonging therapies before ^177^Lu-PSMA. Common Terminology Criteria for Adverse Events grade 3–4 treatment-emergent adverse events during ^177^Lu-PSMA therapy and a 30-d follow-up period included anemia (18%) and thrombocytopenia (2%). Median overall survival was 12.6 mo (95% CI, 8.8–16.1 mo) and 31.4 mo (95% CI, 25.7–37.6 mo) from starting ^177^Lu-PSMA or ^223^Ra, respectively. **Conclusion:** ^177^Lu-PSMA treatment was well tolerated in patients who had received prior ^223^Ra. ^223^Ra use before ^177^Lu-PSMA is feasible and can be considered for future assessment of the optimal treatment sequence.

## Visual Abstract

**Figure:**
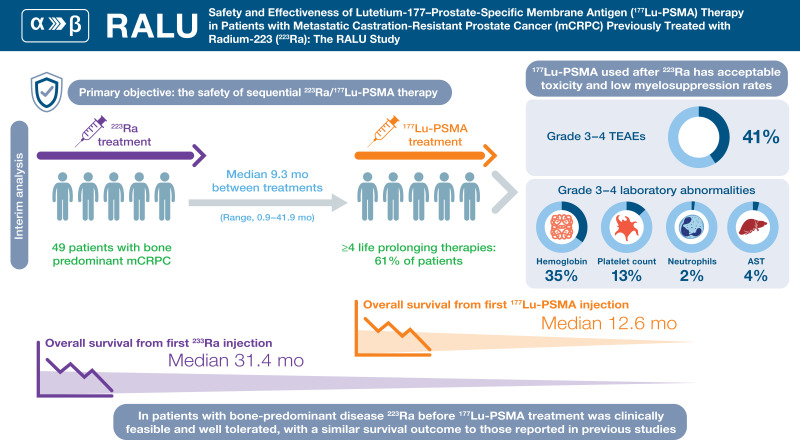

Overall survival and quality of life in patients with bone-predominant metastatic castration-resistant prostate cancer (mCRPC) was improved by ^223^Ra-dichloride, a targeted α-therapy with a good safety profile (1). ^223^Ra therapy results in low myelosuppression rates, and recent preclinical data demonstrated its transient effect on the bone marrow without long-term effects (1*,*^2^). Therefore, earlier incorporation of ^223^Ra in the treatment sequence may facilitate optimal build-in of life-prolonging therapies to improve survival outcomes.

The VISION study investigated a β-emitter, ^177^Lu-PSMA-617, targeting PSMA-expressing cells and found prolonged overall survival and acceptable safety in heavily pretreated patients with mCRPC (3). Another ^177^Lu-PSMA radioligand (^177^Lu-PSMA-I&T) was also well tolerated, with few hematologic adverse events (AEs) of grade 3 or higher (4).

^223^Ra and ^177^Lu-PSMA regulatory approval (in some countries) for patients with mCRPC, albeit in different patient populations, prompted us to investigate the safety and survival outcomes of sequential ^223^Ra and ^177^Lu-PSMA. In VISION, 17.4% of patients received ^223^Ra therapy before ^177^Lu-PSMA without adversely affecting efficacy, but safety has not been reported for this subgroup (5). However, retrospective studies have shown that using ^223^Ra before ^177^Lu-PSMA is feasible, with acceptable safety (6*,*^7^). Moreover, ^177^Lu-PSMA-617 initiation at no more than 8 wk after ^223^Ra in patients with progressive bone-metastatic disease was effective, with acceptable safety (8). We analyzed interim data from the observational radium lutetium (RALU) study to further evaluate safety and survival for sequential ^223^Ra and ^177^Lu-PSMA therapy in patients with mCRPC.

## MATERIALS AND METHODS

The RALU study was a retrospective, multicenter medical chart review investigating the safety of ^177^Lu-PSMA in patients with mCRPC previously treated with ^223^Ra. This analysis includes patients treated in Germany. Patients were at least 18 y old with mCRPC and received at least 1 ^223^Ra injection and subsequently at least 1 ^177^Lu-PSMA cycle.

**FIGURE 1.**
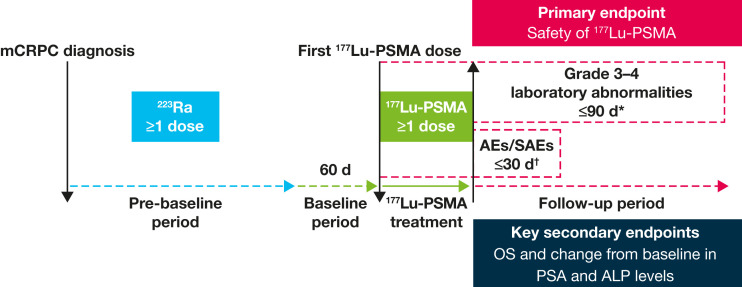
RALU study design. *From ^177^Lu-PSMA start to 90 d after last dose. ^†^From ^177^Lu-PSMA start to 30 d after last dose. ALP = alkaline phosphatase; OS = overall survival; PSA = prostate-specific antigen; SAEs = serious AEs.

The retrospective observation period started at mCRPC diagnosis and ended either at the last available visit or death, whichever occurred first. Prebaseline, baseline, and follow-up period definitions are shown in Figure 1.

The primary endpoint was the safety of ^177^Lu-PSMA after ^223^Ra therapy. AEs used Common Terminology Criteria for Adverse Events grading. Secondary endpoints included OS, time to next treatment, and change from baseline in serum prostate-specific antigen and alkaline phosphatase levels. AEs and grade 3–4 laboratory abnormalities were recorded as per Figure 1.

The study was conducted in accordance with relevant guidelines and regulations (supplemental methods).

## RESULTS

**FIGURE 2.**
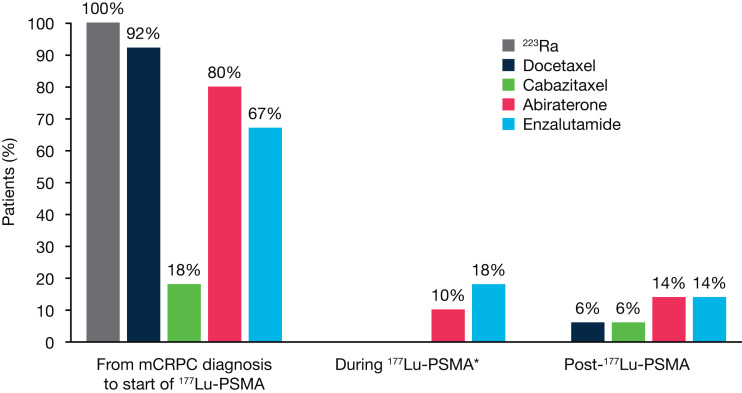
Use of life-prolonging therapies. *Chemotherapy was not used concomitantly with ^177^Lu-PSMA.

This preplanned interim analysis included medical records from 49 patients (data cutoff, January 31, 2022) (Table 1). Before ^177^Lu-PSMA initiation, 31% (15/49) of patients had visceral metastases, and median prostate-specific antigen and alkaline phosphatase values were 287.0 ng/mL and 142.5 U/L, respectively (Table 1). At least 1 line of taxane-based chemotherapy was received by 92% (45/49) of patients before ^177^Lu-PSMA initiation, with 51% (25/49) receiving taxane-based chemotherapy between ^223^Ra and ^177^Lu-PSMA (Table 1). Before starting ^177^Lu-PSMA, 63% (30/49) of patients received at least 4 life-prolonging therapies (docetaxel, cabazitaxel, abiraterone, enzalutamide, and ^223^Ra; Table 1; Fig. 2). Most patients received chemotherapy before ^177^Lu-PSMA; 92% received docetaxel, and 18% received cabazitaxel (Fig. 2).

**TABLE 1.** Baseline Characteristics Before Starting ^177^Lu-PSMA

Characteristic	Data
Total patients	49 (100)
Age (y)	72 (57–83)
Eastern Cooperative Oncology Group performance status (baseline)	
0	0 (0)
1	36 (73)
2	13 (27)
3–4	0 (0)
Prostate-specific antigen (ng/mL)	287.0 (20–12,229)
Alkaline phosphatase (U/L)	142.5 (48–730)
Visceral metastatic disease	15 (31)
≥4 life-prolonging therapies*	30 (61)
Novel antiandrogen therapies	
Abiraterone	39 (80)
Enzalutamide	33 (67)
Abiraterone and enzalutamide	33 (67)
Number of any taxane-based chemotherapy lines^†^	
0	4 (8)
1	35 (71)
≥2	10 (20)
Docetaxel	45 (92)
Number of docetaxel cycles^‡^	
1–4	10 (20)
≥5	26 (53)
Cabazitaxel	9 (18)
Number of cabazitaxel cycles^‡^	
1–4 cycles	0 (0)
≥5 cycles	5 (10)
Taxane-based chemotherapy between ^223^Ra and ^177^Lu-PSMA^§^	25 (51)

* Docetaxel, cabazitaxel, abiraterone, enzalutamide, and ^223^Ra.

† Chemotherapies with same start date ± 15 d are counted as 1 line.

‡ Not available for some patients.

§ After last ^223^Ra dose and 60 d before ^177^Lu-PSMA.

Qualitative data are number and percentage; continuous data are median and range.

The median time from the first ^223^Ra and ^177^Lu-PSMA dose to the end of observation was 28.0 mo (range, 11.0–68.2 mo) and 8.9 mo (range, 1.4–63.9 mo), respectively. A median of 6 ^223^Ra injections was administered, and 71% (35/49) of patients received 5–6 ^223^Ra injections. The median ^223^Ra therapy duration was 4.7 mo (range, 1.0–7.0 mo). All patients received ^177^Lu-PSMA (^177^Lu-PSMA-617 [67%] or ^177^Lu-PSMA-I&T [33%]), and 59% (29/49) received at least 4 ^177^Lu-PSMA cycles. The median ^177^Lu-PSMA therapy duration was 4.9 mo (range, 0–57.1 mo). The median time from the last ^223^Ra injection to the first ^177^Lu-PSMA dose was 9.3 mo (range, 0.9–41.9 mo). Overall, 51% (25/59) of patients received taxane-based chemotherapy during or after ^223^Ra and until 60 d before ^177^Lu-PSMA.

During ^177^Lu-PSMA, 92% (45/49) of patients experienced any grade of treatment-emergent AEs and 41% (20/49) experienced grade 3–4 (Supplemental Table 1). Grade 3–4 anemia and thrombocytopenia occurred in 18% (9/49) and 2% (1/49) of patients, respectively. Grade 1–2 dry mouth occurred in 27% (13/49) of patients; none had grade 3–4 dry mouth. One patient (2%) had grade 1–2 dry eye. The incidence of grade 3–4 laboratory abnormalities was highest for anemia (35% [17/49]) and thrombocytopenia (13% [6/49]) (Table 2). No grade 5 toxicities occurred.

**TABLE 2.** Incidence of Grade 3–4 Laboratory Abnormalities Measured from ^177^Lu-PSMA Start to 90 Days After Last Dose

Abnormality	Patients (*n*)	Incidence (*n*)
Hemoglobin	49	17 (35%)
Platelet count	47	6 (13%)*
Neutrophils	49	1 (2%)
Aspartate aminotransferase	49	2 (4%)

* Four of 6 had low platelets at baseline, with further reductions at follow-up (1 had chemotherapy before ^177^Lu-PSMA); 1 of 6 had normal platelets at baseline, with reductions seen after chemotherapy and at follow-up; 1 of 6 had normal platelets at baseline, with reduction at follow-up.

**FIGURE 3.**
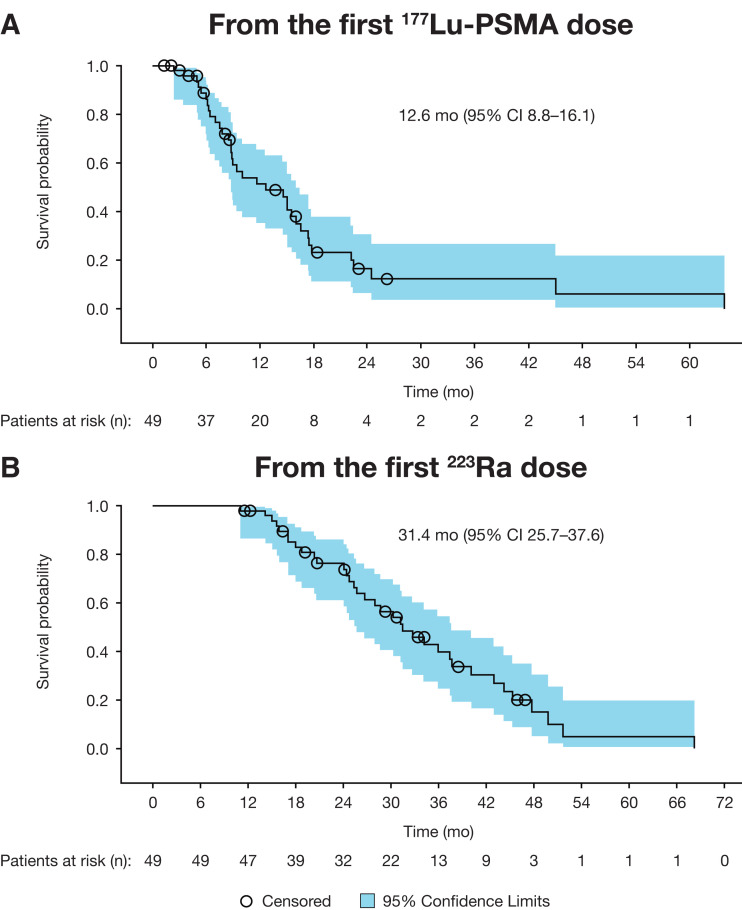
Kaplan–Meier plots for overall survival calculated from first ^177^Lu-PSMA (A) and ^223^Ra (B) dose.

Median overall survival was 12.6 mo (95% CI, 8.8–16.1 mo) and 31.4 mo (95% CI, 25.7–37.6 mo) from the first dose of ^177^Lu-PSMA and ^223^Ra, respectively (Fig. 3). During ^177^Lu-PSMA, 39% and 29% of patients had at least a 30% or 50% decline in prostate-specific antigen (best response), respectively; corresponding alkaline phosphatase declines were 6% and 4%.

## DISCUSSION

Randomized trials have demonstrated low myelosuppression rates in patients with mCRPC receiving ^223^Ra or ^177^Lu-PSMA (1*,*^3^*,*^9^). However, chemotherapy and advanced disease affecting bone marrow function can increase myelosuppression rates in this setting (10–12). Therefore, in real-world practice, radiopharmaceutical therapy after prior chemotherapy may result in more serious hematologic AEs.

^177^Lu-PSMA after ^223^Ra treatment had an acceptable safety profile. Notably, this was despite the heavy pretreatment of the patient population, with more than 90% of patients having received chemotherapy in addition to ^223^Ra and ^177^Lu-PSMA. Grade 3–4 anemia and thrombocytopenia incidences were 18% and 2%, respectively, consistent with the retrospective analysis of patients receiving the ^223^Ra and ^177^Lu-PSMA sequence in the real-world REASSURE study (15% and 4%, respectively) (6). When ^177^Lu-PSMA was given within 8 wk of ^223^Ra, the incidence of anemia of at least grade 3 was similar (18%), but the rates of leukopenia and thrombocytopenia of at least grade 3 were higher than reported here (14% vs. 0 and 21% vs. 2%, respectively) (8).

The median overall survival from the start of ^177^Lu-PSMA or ^223^Ra therapy (12.6 and 31.4 mo, respectively) corresponded to that reported in REASSURE (13.2 and 28.0 mo, respectively) (6). In patients with mCRPC who underwent ^177^Lu-PSMA therapy in the WARMTH study, overall survival was longer in patients with bone metastases receiving prior ^223^Ra than in those who did not (16 vs. 12 mo in patients with 6–20 bone lesions, *P* = 0.038, and 11 vs. 7 mo in patients with diffuse involvement, *P* = 0.034) (12).

This study’s strength is underlined by broad inclusion criteria and high-quality data with few missing datapoints. Accordingly, we could effectively evaluate ^177^Lu-PSMA safety in patients with a history of ^223^Ra therapy who received chemotherapy, before or after ^223^Ra treatment. Nevertheless, a retrospective study design may have contributed to a patient selection bias due to the preset outcomes of interest. Other limitations include retrospective AE grading, lack of ascertainment of ^177^Lu-PSMA doses and schedules, and a lack of comparison to patients who were not pretreated with ^223^Ra. Despite small patient numbers, patients were managed and treated in high-volume German nuclear medicine centers with extensive ^223^Ra and ^177^Lu-PSMA experience.

## CONCLUSION

This retrospective cohort study demonstrated that, for patients with bone-predominant mCRPC who were receiving ^223^Ra in routine care, subsequent ^177^Lu-PSMA treatment was clinically feasible and well tolerated, with limited myelosuppression. Survival outcomes reflected those of previous reports. Therefore, in patients with bone-predominant mCRPC, ^223^Ra use before ^177^Lu-PSMA can be considered in future assessments of the optimal sequence for life-prolonging therapies.

## DISCLOSURE

Cancer Communications and Consultancy Ltd., Cheshire, U.K., provided medical writing assistance (funded by Bayer). Dr. Lila Adnane (Bayer) provided editorial assistance. Kambiz Rahbar receives honoraria from Advanced Accelerator Applications (AAA) and Bayer and has a consultancy/advisory role with ABX GmbH, ABX-CRO, Bayer, and AAA. Markus Essler has a consultancy/advisory role with Bayer, AAA, and Ipsen and receives travel funds from Ipsen. Matthias Eiber owns stocks or has other ownership interests in Novartis and Telix Pharmaceuticals; has a consultancy/advisory role with Blue Earth Diagnostics, ABX Advanced Biochemical Compounds, Janssen Oncology, Telix Pharmaceuticals, and Novartis; receives research funding from Siemens, ABX Advanced Biochemical Compounds, Blue Earth Diagnostics, and Bayer; has a patent application for rhPSMA; and receives travel funds from Bayer Schering Pharma. Christian la Fougère has a consultancy/advisory role with Novartis, EUSA-Pharma, Ipsen, Oncodesign, and Sirtex Medical and receives research funding from Oncovision. Vikas Prasad receives honoraria from AAA; has a consultancy/advisory role with Bayer; and receives research funding from Ipsen. Wolfgang Fendler receives honoraria from Parexel and AAA; has a consultancy/advisory role with Janssen, Calyx, and Bayer; and receives research funding from SOFIE. Philipp Rassek is an employee of Porterhouse Group AG Paracelsus Kliniken. Helmut Dittmann has a consultancy/advisory role with Bayer, Ipsen, and Eisai AG. Ralph Bundschuh receives honoraria from Eisai AG and has a consultancy/advisory role with Bayer. Kim Pabst receives a Junior Clinician Scientist Stipend from the University Medicine Essen Clinician Scientist Academy (sponsor: Faculty of Medicine and Deutsche Forschungsgemeinschaft) and research funding from Bayer. Milena Kurtinecz, and Frank Verholen are employees of Bayer. Oliver Sartor has a consultancy/advisory role with Bayer, Sanofi, AstraZeneca, Dendreon, Constellation Pharmaceuticals, AAA, Pfizer, Bristol-Myers Squibb, Bavarian Nordic, EMD Serono, Astellas Pharma, Progenics, Blue Earth Diagnostics, Myovant Sciences, Myriad Genetics, Novartis, Clarity Pharmaceuticals, Fusion Pharmaceuticals, Isotopen Technologien, Janssen, Noxopharm, Clovis Oncology, Noria Therapeutics, Point Biopharma, TeneoBio, Telix Pharmaceuticals, and Theragnostics; receives travel funds from Bayer, Johnson & Johnson, Sanofi, AstraZeneca, and Progenics; provides expert testimony for Sanofi; owns stocks or has other ownership interests in Lilly, GlaxoSmithKline, Abbvie, Cardinal Health, United Health Group, PSMA Therapeutics, Clarity Pharmaceuticals, Noria Therapeutics, and Clovis Oncology; and receives research funding from Bayer, Sanofi, Endocyte, Merck, InVitae, Constellation Pharmaceuticals, AAA, AstraZeneca, Dendreon, SOTIO, Janssen, and Progenics. No other potential conflict of interest relevant to this article was reported.

## Notes

KEY POINTS **QUESTION:** Is it safe to use ^177^Lu-PSMA to treat patients with mCRPC if they have previously received ^223^Ra? **PERTINENT FINDINGS:** Low rates of overall and hematologic AEs indicated an acceptable safety profile for this treatment sequence. Median OS was 12.6 and 31.4 mo from the first dose of ^177^Lu-PSMA and ^223^Ra, respectively, and 39% of patients had at least a 30% decline in prostate-specific antigen. **IMPLICATIONS FOR PATIENT CARE:** Introduction of ^223^Ra early in the treatment sequence in patients with bone-predominant mCRPC and subsequent treatment with ^177^Lu-PSMA is feasible, well tolerated, and effective.

## References

[1] Parker C Nilsson S Heinrich D. Alpha emitter radium-223 and survival in metastatic prostate cancer. N Engl J Med. 2013;369:213–223. 23863050 10.1056/NEJMoa1213755

[2] Parker CC Coleman RE Sartor O. Three-year safety of radium-223 dichloride in patients with castration-resistant prostate cancer and symptomatic bone metastases from phase 3 randomized alpharadin in symptomatic prostate cancer trial. Eur Urol. 2018;73:427–435. 28705540 10.1016/j.eururo.2017.06.021

[3] Sartor O de Bono J Chi KN. Lutetium-177-PSMA-617 for metastatic castration-resistant prostate cancer. N Engl J Med. 2021;385:1091–1103. 34161051 10.1056/NEJMoa2107322 PMC8446332

[4] Heck MM Tauber R Schwaiger S. Treatment outcome, toxicity, and predictive factors for radioligand therapy with ^177^Lu-PSMA-I&T in metastatic castration-resistant prostate cancer. Eur Urol. 2019;75:920–926. 30473431 10.1016/j.eururo.2018.11.016

[5] Vaishampayan N Morris MJ Krause BJ. [^177^Lu]Lu-PSMA-617 in PSMA-positive metastatic castration-resistant prostate cancer: prior and concomitant treatment subgroup analyses of the VISION trial. J Clin Oncol. 2022;40(16_Suppl):5001.

[6] Sartor O la Fougere C Essler M. Lutetium-177-prostate-specific membrane antigen ligand after radium-223 treatment in men with bone-metastatic castration-resistant prostate cancer: real-world clinical experience. J Nucl Med. 2021;63:410–414. 34168015 10.2967/jnumed.121.262240 PMC8978191

[7] Ahmadzadehfar H Zimbelmann S Yordanova A. Radioligand therapy of metastatic prostate cancer using ^177^Lu-PSMA-617 after radiation exposure to ^223^Ra-dichloride. Oncotarget. 2017;8:55567–55574. 28903443 10.18632/oncotarget.15698 PMC5589682

[8] Baumgarten J Groener D Nguyen Ngoc C. Safety and efficacy of ^177^lutetium-PSMA-617 radioligand therapy shortly after failing ^223^radium-dichloride. Cancers (Basel). 2022;14:557. 35158825 10.3390/cancers14030557 PMC8833613

[9] Hofman MS Emmett L Sandhu S. [^177^Lu]Lu-PSMA-617 versus cabazitaxel in patients with metastatic castration-resistant prostate cancer (TheraP): a randomised, open-label, phase 2 trial. Lancet. 2021;397:797–804. 33581798 10.1016/S0140-6736(21)00237-3

[10] Tannock IF de Wit R Berry WR. Docetaxel plus prednisone or mitoxantrone plus prednisone for advanced prostate cancer. N Engl J Med. 2004;351:1502–1512. 15470213 10.1056/NEJMoa040720

[11] de Bono JS Oudard S Ozguroglu M. Prednisone plus cabazitaxel or mitoxantrone for metastatic castration-resistant prostate cancer progressing after docetaxel treatment: a randomised open-label trial. Lancet. 2010;376:1147–1154. 20888992 10.1016/S0140-6736(10)61389-X

[12] Ahmadzadehfar H Matern R Baum RP. The impact of the extent of the bone involvement on overall survival and toxicity in mCRPC patients receiving [^177^Lu]Lu-PSMA-617: a WARMTH multicentre study. Eur J Nucl Med Mol Imaging. 2021;48:4067–4076. 34031719 10.1007/s00259-021-05383-3

